# 1932. Molecular Epidemiology of NDM-producing *Acinetobacter baumannii* in the US—October 2013—March 2022

**DOI:** 10.1093/ofid/ofad500.092

**Published:** 2023-11-27

**Authors:** Magdalena Medrzycki, Richard A Stanton, Danielle A Rankin, Sam Horwich-Scholefield, Arif Mahmud, Tyler Maruca, Sarah Brister, Lian Hsiao, Elisabeth Vaeth, Michelle Therrien, Erin L Young, Kelly F Oakeson, Farhana Haque, Lindsay Neff, Alison L Halpin, Michael Tran, Kirstin L Veliz, Chi Hua, Emily C Schneider, Walters Maroya

**Affiliations:** CDC, Atlanta, GA; Division of Healthcare Quality Promotion, Centers for Disease Control and Prevention, Atlanta, GA; Centers for Disease Control and Prevention, Atlanta, Georgia; California Department of Public Health, Richmond, CA; Texas Department of State Health Services, Austin, Texas; Maryland Department of Health, Baltimore, Maryland; Illinois Department of Public Health, Chicago, Illinois; California Department of Public Health, Healthcare-Associated Infections Program, Berkeley, California; Maryland Department of Health, Baltimore, Maryland, Baltimore, Maryland; Antibiotic Resistance Laboratory Network (ARLN), Nashville, Tennessee; Department of Health and Human Services, Public Health Laboratory, Salt Lake City, Utah; Utah Department of Health & Human Services / Utah Public Health Laboratory, Taylorsville, Utah; Maryland Department of Health, Baltimore, Maryland; Utah Public Health Laboratory, West Valley City, Utah; Centers for Disease Control and Prevention, Atlanta, Georgia; Washington State Department of Health, Shoreline, Washington; WA State Department of Health, Shoreline, Washington; WA Public Health Laboratories, Shoreline, Washington; Washington State Department of Health, Shoreline, Washington; CDC, Atlanta, GA

## Abstract

**Background:**

In the United States, most carbapenem-resistant *Acinetobacter baumannii* (CRAB) produce carbapenem-hydrolyzing class D β-lactamases (OXA); other carbapenemase classes have historically been uncommon. We describe the epidemiology and molecular characteristics of New Delhi Metallo-β-lactamase (NDM)-producing CRAB reported to CDC since October 2013.

**Figure 1:**
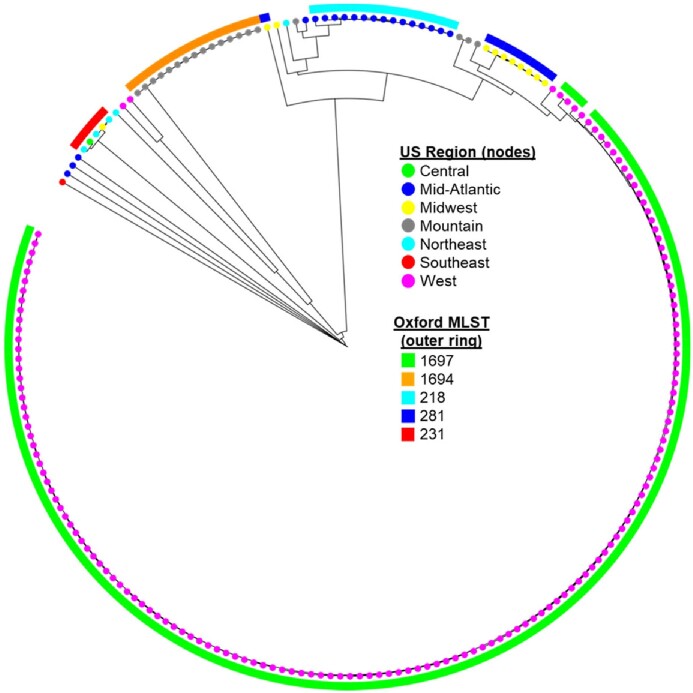
A core genome multilocus sequence typing (cgMLST) scheme phylogenetic tree of 208 the New Delhi Metallo-β-lactamase (NDM), sequenced by US public health laboratories and with publicly available sequences, with dates of collection between November 1, 2018 – March 31, 2022. Four regional clusters were identified in Mountain, Mid-Atlantic, Midwest and West regions. Isolates within each cluster were closely related to each other.

**Figure:**
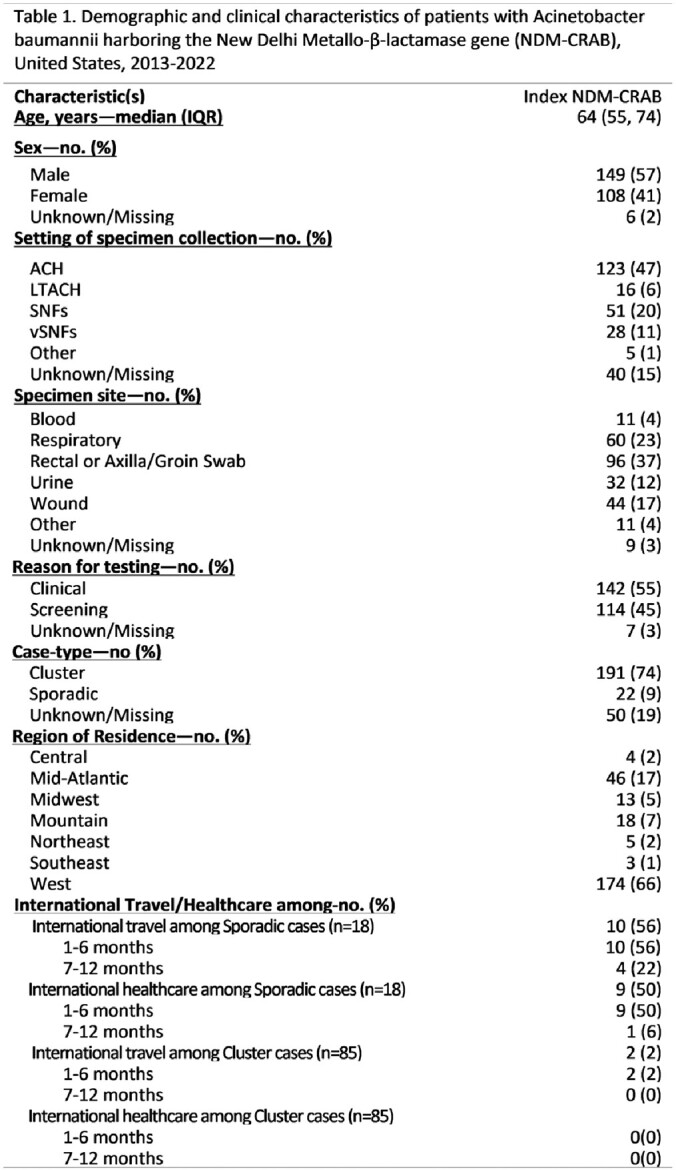

**Methods:**

We included a patient’s first NDM-CRAB isolate reported to CDC through reference antimicrobial susceptibility testing, outbreak response, or Antimicrobial Resistance Laboratory Network, with specimen collection dates during 10/1/2013–3/31/2022. Two or more NDM-CRAB linked in space and time were classified as cluster-associated; other NDM-CRAB as sporadic. We described patient demographics and clinical characteristics and analyzed whole genome sequence (WGS) data for relatedness using both traditional multilocus sequence typing (MLST) with the Oxford scheme (ST_OX_) and core genome (cg) MLST. We compared publicly available sequences of US NDM-CRAB to NDM-CRAB from non-US locations.

**Results:**

CDC received reports of 263 NDM-CRAB from unique patients in 21 states. Patients had a median age of 65 years (IQR: 55-74) (Table 1). NDM-CRAB were from clinical cultures of respiratory (60), wound (44), urine (32), blood (11), and other sites (11) and from rectal or axilla/groin cultures (96). Eleven of 103 patients with information available were hospitalized outside the US ≤12 months prior to NDM-CRAB specimen collection, 82% were sporadic cases and 19% were a part of clusters. Overall, 191 were from cluster-associated cases in four distinct geographic regions that reported multifacility outbreaks; all cluster-associated cases were from cultures collected after June 15, 2019. WGS data were available for 208 (79%) isolates. The four regional outbreaks identified in the epidemiologic investigation were different ST_OX_ (Fig. 1); three (ST218, ST281, and ST1697) were closely related to NDM-CRAB isolates from outside the US.

**Conclusion:**

NDM-CRAB reported to CDC appears to reflect domestic acquisition rather than importation. ST differences across the regional outbreaks indicate they arose from unique introductions rather than inter-regional spread or dissemination of a successful clone.

**Disclosures:**

**All Authors**: No reported disclosures

